# Maslinic Acid, a Natural Phytoalexin-Type Triterpene from Olives — A Promising Nutraceutical?

**DOI:** 10.3390/molecules190811538

**Published:** 2014-08-04

**Authors:** Glòria Lozano-Mena, Marta Sánchez-González, M. Emília Juan, Joana M. Planas

**Affiliations:** Departament de Fisiologia and Institut de Recerca en Nutrició i Seguretat Alimentària (INSA-UB), Universitat de Barcelona (UB), Av. Joan XXIII s/n, 08028 Barcelona, Spain

**Keywords:** maslinic acid, phytochemical, phytoalexin, pentacyclic triterpene, biological effects, antitumor, antidiabetic, antioxidant, nutraceutical

## Abstract

Maslinic acid is a pentacyclic triterpene found in a variety of natural sources, ranging from herbal remedies used in traditional Asian medicine to edible vegetables and fruits present in the Mediterranean diet. In recent years, several studies have proved that maslinic acid exerts a wide range of biological activities, *i.e.* antitumor, antidiabetic, antioxidant, cardioprotective, neuroprotective, antiparasitic and growth-stimulating. Experimental models used for the assessment of maslinic acid effects include established cell lines, which have been often used to elucidate the underlying mechanisms of action, and also animal models of different disorders, which have confirmed the effects of the triterpene *in vivo*. Overall, and supported by the lack of adverse effects in mice, the results provide evidence of the potential of maslinic acid as a nutraceutical, not only for health promotion, but also as a therapeutic adjuvant in the treatment of several disorders.

## 1. Introduction

**Figure 1:**
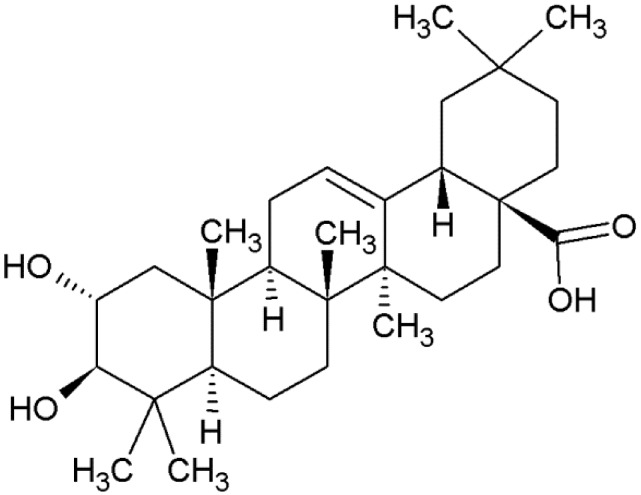
Chemical structure of maslinic acid.

Maslinic acid, also known as crategolic acid or (2α,3β)-2,3-dihydroxyolean-12-en-28-oic acid (Figure 1), is a pentacyclic triterpene widely distributed in the plant kingdom. In the last decades, and in response to an increasing interest to identify new natural molecules with beneficial effects on health, maslinic acid has been isolated not only from various plants used in traditional herbal medicine, but also from edible vegetables and fruits. In parallel, the biological activities of maslinic acid have been assessed in different experimental models, from tumor cell lines to animal models of several diseases, supported by the lack of adverse effects *in vivo* after the oral administration of the triterpene [1]. In summary, maslinic acid is arising as a novel natural and safe molecule with different biological targets, which might derive to considering it as a nutraceutical in the future.

Historically, maslinic acid was named “crategolic acid”, since it was first isolated from *Crataegus oxyacantha* L. [2] Tschesche *et al.* [3] described it as a triterpenoid carboxylic acid with molecular formula C_30_H_48_O_4_, mainly found in the leaves of the abovementioned species, where it accounted for 25%−30% of the amount of triterpenoids in this tissue [4]. In the early 1960s, a series of studies by other authors reported the identification of a new triterpenic acid from *Olea europaea* L., although with some controversy. Caglioti *et al.* [5] isolated from olive husks a triterpenic acid with molecular formula and structure identical to those of crategolic acid, and named it maslinic acid. However, a few years later the study was questioned, since the results could not be reproduced, and maslinic acid was considered a product derived from the aging of the fruit [6]. In parallel to the work by Caglioti *et al.* [5], Vioque and Morris [7] found two triterpenic acids in the acetonic extract of the olive pomace, one of which was identified as oleanolic acid and the other was defined as a dihydroxytriterpenic acid, which could be maslinic acid. More than three decades later, Bianchi *et al.* [8] shed light about the composition of the olive fruit, quantifying maslinic acid together with oleanolic acid as the major lipidic compounds in the cuticle of the drupe.

### 1.1. Biosynthesis and Role as a Phytoalexin

Triterpenoids, such as maslinic acid, are a group of secondary metabolites derived from the cyclation of squalene, oxidosqualene or bis-oxidosqualene [9]. These precursors (C_30_) are substrate of several types of triterpene synthases, which catalyze their cyclation through intermediate cations to a wide variety of triterpenes. Depending on the number of rings, the latter are classified as mono-, bi-, tri-, tetra- or pentacyclic triterpene alcohols [9]. Lupeol, α- and β-amyrin are examples of pentacyclic triterpene alcohols, which not only constitute secondary metabolites themselves, but also might undergo oxidation reactions to yield other derivatives, such as betulinic, ursolic and maslinic acids.

Not long after the identification in *Crataegus oxyacantha* L., Tschesche *et al.* [10] recognized maslinic acid as a derivative of the β-amyrin series, but it was Stiti *et al.* [11] who more recently postulated the biosynthetic pathway that leads to the formation of maslinic acid in the fruits of *Olea europaea* L., one of the main natural sources of this triterpene. The authors suggest that in the developing olive both the sterols (primary metabolites) and the non-steroidal triterpenoids (secondary metabolites) share oxidosqualene as a common precursor. The enzyme β-amyrin synthase catalyzes its cyclation into β-amyrin, and further oxidation steps give rise to the triterpenic dialcohol erythrodiol followed by the hydroxy pentacyclic triterpenic acids oleanolic and maslinic [11].

Regarding the function, plant secondary metabolites are not essential for the growth, development and reproduction of individuals, but might contribute to their survival or give them evolutionary advantages. Phytoalexins are a particular case of secondary metabolites, involved in the protection of the plant against pathogens, and maslinic acid can be considered as such, since different studies have proved its protective activity under adverse conditions. Kombargi *et al.* [12] observed that dipping *Olea europaea* L. fruits in solutions of maslinic acid prevented the oviposition of eggs from females of the olive fruit fly (*Bactrocera oleae*), which is the major insect pest of olives in the Mediterranean countries. Furthermore, the isolated triterpene is toxic after ingestion by rice weevil adults (*Sitophilus oryzae*) [13], a widespread and destructive pest of stored cereals.

### 1.2. Natural Sources

Maslinic acid was first detected in *Crataegus oxyacantha* L., but the growing interest in this triterpene because of its wide range of health-enhancing activities has led to its identification in other natural sources, being present in more than 30 plants worldwide. On one hand, the triterpene has been found in plants used in traditional Asian medicine for the treatment of diverse affections. To mention only a few examples, the leaves of loquat (*Eriobotrya japonica*) [14], which have been used as antitussive and anti-inflammatory for chronic bronchitis, and also as diuretic, digestive and antipyretic [15]; the flowers of *Campsis grandiflora*, employed for female disorders like uterine hemorrhage [16]; the whole plant of *Geum japonicum* [17], used as diuretic [18]; and *Agastache rugosa* [19], for the treatment of anorexia, vomiting and other intestinal disorders [20]. On the other hand, maslinic acid has recently been quantified in edible vegetables, such as table olives [21], spinach and eggplant [22], aromatic herbs like mustard and basil [22,23], legumes such as chickpeas and lentils [24], and to a lesser extent in some fruits like mandarin and pomegranate [25] (Table 1). Therefore, plant-based diets might provide a constant supply of maslinic acid, which could be considered, among many other factors, partly responsible of the health-enhancing properties of these dietary habits.

**Table 1:** Maslinic acid content in edible sources.

	Maslinic Acid (mg/kg Dry Weight)	References
**Table olives**		
Kalamata (plain black)	1318	[21]
Hojiblanca (plain green)	905	[21]
Gordal (plain green)	414	[21]
Manzanilla (plain green)	384	[21]
Cacereña (plain black)	295	[21]
**Fresh vegetables**		
Spinach	1260	[22]
Eggplant	840	[22]
**Aromatic herbs**		
Brown mustard	330	[22]
Leaf mustard	1740	[23]
Basil	350, 320	[22,23]
**Cooked legumes**		
Small lentils	26.3	[24]
Large lentils	39.5	[24]
Chickpeas	61.9	[24]
**Fresh fruits**		
Mandarin	1.18	[25]
Pomegranate	10.8	[25]

## 2. Biological Effects

### 2.1. Maslinic Acid and Cancer

The antitumor activity of maslinic acid has become remarkable in recent years, as evidenced by the higher number of studies that address this issue, compared to those about other biological effects. The vast majority of published references correspond to *in vitro* experiments that show the anti-proliferative and/or pro-apoptotic effect of maslinic acid, together with plausible mechanisms of action that involve different signaling pathways. Colon cancer cell lines have been extensively used with this aim, but there is no shortage of studies that prove the above-mentioned effects in a wide range of cell lines from other origins. Moreover, this antitumor effect has also been assessed in several animal models, with positive results that reinforce its potential as anticarcinogenic agent.

#### 2.1.1. Maslinic Acid Exerts an Anti-Proliferative Activity through Arresting Cell Cycle and Activates Both the Intrinsic and the Extrinsic Apoptotic Pathways *in Vitro*

The study conducted by Juan *et al.* [26] demonstrated for the first time the potent anti-proliferative activity of maslinic in the human colorectal adenocarcinoma cell line HT-29. The triterpene did not show non-specific cytotoxicity up to 250 μM, but exerted a dose-dependent anti-proliferative activity with IC_50_ of 101.2 μM at 72 h of exposure [27]. Similar results were found by Reyes *et al.* [28] in both the colon cancer cell line HT-29 and Caco-2, in which incubation with the triterpene for 72 h resulted in inhibition of cell growth with IC_50_ of 61 μM and 85 μM, respectively. Further experiments by the same authors revealed that maslinic acid exerted its anti-proliferative activity by arresting cell cycle, since the cell population in the G0/G1 phases was significantly increased, while that in the S phase was reduced [28]. Remarkably, in both studies the effect of the compound on cell proliferation coincided with apoptotic cell death.

Apoptosis, also called programmed cell death, refers to a cascade of biochemical events that lead to the disintegration of the cell into fragments, which are further removed by phagocytic cells without eliciting an inflammatory response. This process might occur through death receptors, the so-called extrinsic pathway, or by means of an intrinsic pathway, in which mitochondria play a role. Both routes converge at the level of caspase-3, which is one of the effector caspases [29]. Interestingly, maslinic acid has been found to affect both pathways at different levels.

In the study of Juan *et al.* [27], the activation of caspase-3 was more than 60-fold at 24 h of exposure to 250 μM of the triterpene, compared to vehicle-treated cells. In order to know whether the activation of caspase-3 resulted from the extrinsic or the intrinsic pathway, the production of superoxide anions was evaluated, since it is one of the possible inductors of the latter [30]. Indeed, higher levels of O_2_^−^ were found in cells incubated with maslinic acid (150 μM) for 4 h, compared to controls. The apoptotic process was further confirmed by the occurrence of plasma membrane disintegration and nuclear fragmentation [27]. Similarly, Reyes *et al.* [28] also reported that the apoptotic process observed in both HT-29 and Caco-2 cell lines occurred through activation of caspase-3, as evidenced by the observation of morphological changes, such as cell shrinkage or chromatin condensation.

Attention was then drawn to the molecular events underlying the induction of the mitochondrial apoptotic pathway. This organelle is a reservoir of several pro-apoptotic proteins that upon the proper stimulus are released to the cytosol, where the interaction with other elements finally triggers caspase-3 activation. An important set of regulators of this pathway is the Bcl-2 family, which includes both anti- and pro-apoptotic members [29].

Experiments performed by Reyes-Zurita *et al.* [31] with HT-29 cells showed that maslinic acid concomitantly activated the expression of Bax (pro-apoptotic protein) and inhibited the expression of Bcl-2 (anti-apoptotic protein), resulting in mitochondrial disruption and cytochrome-c release to the cytosol. It is known that once in the cytosol cytochrome-c binds to Apaf-1, which triggers the sequential activation of caspase-9 and caspase-3 [32]. Although in this study the formation of the complex was not directly assayed, a strong time- and dose-dependent cleavage of both caspases was observed [31].

More recently, the same authors postulated that the effect of maslinic acid on Bcl-2 family proteins could be mediated by the kinase JNK, since its expression was found increased in HT-29 cells after a 12 h treatment with the triterpene [33]. Actually, some of the effects of JNK had been previously described. Tsuruta *et al.* [34] found that JNK promotes Bax translocation to mitochondria through phosphorylation (inactivation) of a cytoplasmic anchor of Bax. Another consequence of JNK activation is the cleavage of Bid (pro-apoptotic protein), which results in translocation to mitochondria and Smac/DIABLO release to the cytosol [35]. This protein induces apoptosis through neutralizing inhibitors of apoptosis (IAPs) [36]. Apart from JNK activation, maslinic acid also enhanced the expression of p53, which is a well-known tumor-suppressor transcription factor that regulates the expression of genes involved in apoptosis, such as those coding for the above-mentioned Bcl-2 and Bax proteins [37].

In contrast with the intrinsic pathway, the extrinsic route is initiated by the binding of a ligand with a receptor of the tumor necrosis factor receptor (TNFR) superfamily. This results in the assembly of several elements, which constitute the so-called complex I [38]. Next, two possible ways trigger the regulation of apoptosis with opposite outcomes. On one hand, complex I can activate the kinase IKK, responsible of the phosphorylation of IKBα and its subsequent degradation. IKBα normally recruits NF-κB in the cytosol, but after its degradation the transcription factor is released and translocates to the nucleus [39], where it up-regulates anti-apoptotic genes [40]. On the other hand, some elements of the complex I can be exchanged, including the recruitment of procaspase-8, and this leads to the formation of a secondary complex (complex II) [38]. Activation of procaspase-8 results in the cleavage of downstream effector caspases, such as caspase-3, thus propagating the apoptotic signal [41].

The role of maslinic acid in the death-receptor pathway was first demonstrated by Li *et al.* [42] using the pancreatic cancer cells Panc-28. The compound exerted a synergistic effect together with TNF-α on both inhibition of cell proliferation (maslinic acid at 10 μM) and induction of cell death (25 μM), being the latter more than 55% higher, compared to control. The determination of activated caspase-3 in the cells confirmed the occurrence of apoptosis. Further experiments showed that maslinic acid affected the NF-κB pathway by inhibiting IKBα phosphorylation, thus preventing both NF-κB translocation to nucleus and its DNA binding activity.

The inhibitory effect of maslinic acid on NF-κB DNA-binding activity was also proved in the Raji B lymphoma cell line [43]. In this study, the impaired function of NF-κB was used to explain the dose-dependent reduction of COX-2 expression. COX-2 is well-known for its role in the inflammatory process and has been found overexpressed in a wide range of premalignant and malignant tissues [44].

The NF-κB transcriptional activity can be modulated through phosphorylation by various members of the mitogen-activated protein kinase family (MAPK), including JNK and p38 [45]. Wu *et al.* [46] described for the first time that maslinic acid also interacts with the p38 cascade so that ultimately triggers a pro-apoptotic effect. The experiments were performed in two cell lines of human salivary gland adenoid cystic carcinoma, ACC-2 and ACC-M, corresponding to low and high metastasis, respectively. The anti-proliferative activity after 24 h of incubation (IC_50_ of 43.6 and 45.8 μM, respectively) was attributed to an apoptotic process, as evidenced by the observation of both apoptotic bodies and microstructural changes, such as chromosomal DNA condensation and loss of microvilli. Cells exposed to the triterpene showed activated caspase-3, and this occurred as a consequence of p38 MAPK phosphorylation, which in turn was the result of an increase in the concentration of intracellular Ca^2+^. The mechanism by which maslinic acid provokes intracellular Ca^2+^ overload remains to be investigated. On the contrary, the implication of p38 MAPK in maslinic acid-induced apoptosis is consistent with the results obtained in two cell lines of human urinary bladder carcinoma (T24 and 253J) [47]. Incubation with the triterpene dose- and time-dependently increased p38 phosphorylation, and this was correlated with reduced cell survival (IC_50_ of 33.0 and 71.8 μM in each cell line, respectively).

The latest assessment of the anti-proliferative activity of maslinic acid *in vitro* has been performed in the soft tissue sarcoma cell lines SW982 (human synovial sarcoma) and SK-UT-1 (leiomyosarcoma). IC_50_ values were of 45.3 and 59.1 μM, after incubating the cells with the triterpene for 24 h [48]. However, the most remarkable contribution of this study is the fact that maslinic acid is proposed as an adjuvant of the established anticancer drug doxorubicin, which constitutes a novel therapeutic approach for the treatment of cancer diseases. Concretely, cells treated simultaneously with both compounds showed higher sensitivity to doxorubicin as a consequence of an increased intracellular accumulation of the drug. Since doxorubicin is a well-known substrate of the efflux proteins P-gp and MRP1, a plausible mechanism behind the intracellular accumulation of the drug when co-incubated with maslinic acid could be that the triterpene inhibited these transporters. A kinetic study revealed that the parameters V_max_ and K_m_ (obtained by the Michaelis-Menten equation) of P-gp were not affected by maslinic acid, while those of MRP1 were dose-dependently lowered, thus indicating that maslinic behaved as a non-competitive inhibitor of MRP1 [48]. Table 2 summarizes the IC_50_ values of the anti-proliferative activity of maslinic acid found in different cell lines.

**Table 2:** *In vitro* anti-proliferative effect of maslinic acid.

Origin	Cell Line	IC_50_ (μM)	References
Human colorectal adenocarcinoma	HT-29	101.2	[27]
	HT-29	61	[28]
	Caco-2	85	[28]
	Caco-2	15.4	[49]
Human hepatocellular carcinoma	HepG2	69.1	[49]
Human breast adenocarcinoma	MCF-7	136.0	[49]
Human salivary gland adenoid cystic carcinoma	ACC-2 (low metastasis)	43.7	[46]
	ACC-M (high metastasis)	45.8	[46]
Human transitional cell urinary bladder carcinoma	T24	33.0	[47]
	253J	71.8	[47]
	TCCSUP	28.0	[47]
Human transitional cell urinary bladder papilloma	RT4	42.7	[47]
Human synovial sarcoma	SW 982	45.3	[48]
Human uterus leiomyosarcoma	SK-UT-1	59.1	[48]

#### 2.1.2. Maslinic Acid Targets Other Cancer-Related Signaling Pathways

Besides the abnormal cell proliferation occurring in tumor growth, angiogenesis emerges in response to the hypoxic environment within the tumor and constitutes another therapeutic target for cancer diseases. The hypoxia inducible factor-1α (HIF-1α) is one of the pivotal regulators of angiogenesis in response to oxygen deficiency and has been found overexpressed in many human cancers [50]. This factor induces the expression of pro-angiogenic molecules, such as the vascular endothelial growth factor (VEGF) and its receptors [51], among others. The new blood vessels might be used by some cells detached from the primary tumor to reach systemic circulation, thus they would be distributed throughout the organism and are likely to ultimately colonize distant tissues. This process requires the action of proteins that degrade the extracellular matrix, such as matrix metalloproteinases (MMP) and urokinase-type plasminogen activator (uPA), which are secreted as inactive forms by either tumor or stroma cells [52,53]. Concretely, the expression of MMP-2, MMP-9 and uPA may be induced by the above-mentioned HIF-1α [51].

Park *et al.* [54] conducted an exhaustive study about the effect of maslinic acid on the metastatic capacity of the human prostate cancer cell DU145. Treatment with the triterpene resulted in a decrease of both basal and EGF-induced migration of cells in a dose-dependent manner (10−25 μM). This effect was correlated with both MMP and uPA systems; firstly, the triterpene reduced both the secretion of pro-MMP-2 and pro-MMP-9, and also MMP-9 mRNA levels. Secondly, a diminished secretion of pro- and active-uPA was observed, together with decreased uPA activity and mRNA levels, and reduced uPA receptor (uPAR) protein levels. Since MMP and uPA systems are regulated by HIF-1α, it was further assessed whether the effects of maslinic acid observed on the proteases took place through the alteration of HIF-1α levels. It was demonstrated that under hypoxic conditions the triterpene not only counteracted the increased expression of HIF-1α but also inhibited its translocation to the nucleus and decreased its half-life from 11.81 min to 4.96 min [54].

Similar results were obtained in three human liver cancer cell lines (Hep3B, Huh7 and HA227) [22]. In this study, however, the effects of maslinic acid were attributed to the antioxidant effect of the triterpene, since reduced levels of reactive oxygen species (ROS) and nitric oxide (NO) were observed in cells treated with maslinic acid. It had been previously reported that these molecules are natural enhancers of the expression of both HIF-1α and VEGF in cancer cells [55].

#### 2.1.3. The Antitumor Activity of Maslinic Acid also Occurs *in Vivo*

Only a few studies up to now have assessed the antitumor activity of maslinic acid in animal models of cancer disorders, compared to the extensive number of references about its *in vitro* effects and their mechanisms. However, the positive outcomes achieved in all them are encouraging and stimulate further research in this field.

The first *in vivo* approach to the antitumor activity of maslinic acid was performed with athymic nu/nu mice in which xenograft pancreatic cells were implanted [42]. The subcutaneous administration of 10 and 50 mg/kg of the triterpene significantly decreased in a dose-dependent manner both the volume and the weight of the tumors, which in turn showed an increased number of apoptotic cells (from 8% in the control group to 21% and 38% in 10 mg/kg and 50 mg/kg groups, respectively) and a reduced expression of two NF-κB-regulated anti-apoptotic genes, Survivin and Bcl-xl.

More recently, Sánchez-Tena *et al.* [56] assessed the effect of a maslinic acid-enriched diet (100 mg/kg) in Apc^Min/+^ mice, a common animal model of spontaneous intestinal polyposis. Results showed that, after a 6-week treatment period, maslinic acid inhibited the formation of polyps in the small intestine by 45%. Microarray analyses of gene expression profiles suggested that the compound inhibited cell-survival signaling and inflammation pathways.

Finally, bladder cancer has also been targeted by maslinic acid, after implanting T24 and 253J cells in nude mice. Both the size and the weight of the tumors were dose-dependently and significantly reduced in the animals treated with intraperitoneal injections of 20 mg/kg of the triterpene every other day over 35 days [47].

In summary, there is strong evidence that maslinic acid targets a variety of signaling pathways that finally trigger an anticarcinogenic effect, both *in vitro* and *in vivo*. Consequently, maslinic acid is emerging as a potential agent for the treatment of cancer disorders, either alone or in combination with other drugs.

### 2.2. Maslinic Acid and Diabetes

The role of maslinic acid in glucose metabolism has also been extensively studied. Wen *et al.* [57] provided the first evidence of the inhibitory effect of the triterpene on glycogen phosphorylases (GP), which catalyze the first step of glycogen breakdown. In a first *in vitro* assay using GPa (activated form of the enzyme) isolated from rat liver, maslinic acid inhibited the enzyme with an IC_50_ of 99 μM, being 6-fold more potent than caffeine, an established GP inhibitor. Based on this finding, the hypoglycemic activity of the triterpene was evaluated *in vivo*, using a mouse model of diabetes induced by adrenalin, which is known to indirectly stimulate glycogenolysis and thus increase glucose blood concentration. After the oral administration of maslinic acid (100 mg/kg) for 7 days, fasted plasma glucose appeared to be up to 46% lower, compared to animals that had received only the vehicle. Further work of the same authors went into detail about the mechanism of inhibition of maslinic acid on GP. The crystal structure of the complex GPb (inactivated form of the enzyme)-maslinic acid was determined, which revealed that the triterpene binds at the allosteric activator site, where the physiological activator AMP binds [58].

The *in vivo* antidiabetic effect of maslinic acid has been also proved in KK-A^y^ mice [59], an animal model for obesity and Type II non-insulin-dependent diabetes. Single oral administrations of the triterpene at doses of 10 and 30 mg/kg significantly diminished plasma glucose at 2 and 4 h after administration, and at the highest dose the effect was sustained up to 7 h. Similar results were obtained when maslinic acid was given daily for 2 weeks at the same doses, being the reduction in both cases of approximately 30%, with respect to control animals. Furthermore, after the repeated oral administration of 10 and 30 mg/kg of the triterpene, a dose-dependent reduction of plasma insulin levels was observed, as well as a decrease of blood glucose concentrations in the insulin tolerance test, *i.e.*, after the subcutaneous injection of insulin. The latter effect might be attributed to the normalization of plasma adiponectin levels, which was observed in groups treated with both 10 and 30 mg/kg doses [59].

Another animal model commonly used in the study of diabetes is the streptozotocin (STZ)-induced hyperglycemic rats. Khathi *et al.* [60] assessed the effect of maslinic acid (80 mg/kg, p.o.) on postprandial blood glucose in this model, and observed that the co-administration of the triterpene with either sucrose or starch significantly reduced the levels of glucose in plasma up until 120 min, in a similar way to that of acarbose, the positive control. Further research was carried out in order to dilucidate the mechanism by which maslinic acid exerted the hypoglycemic effect. On one hand, treatment with the triterpene reversed the higher expression of SGLT1 and GLUT2 found in diabetic animals compared to controls. These transporters are implicated in the intestinal absorption of glucose, thus their downregulation, which was similar to that produced by the standard drugs insulin and metformin, contributed to diminishing plasma glucose. Similarly, the expression of α-glucosidase and α-amylase, which are carbohydrates hydrolyzing enzymes, was attenuated in the small intestine of STZ-induced diabetic rats [60].

The lowering effect of maslinic acid on blood glucose of STZ-induced diabetic rats was consistent with that observed in a previous study [61], in which the triterpene was administered orally at a dose of 50 mg/kg for 28 days and the reduction of plasma glucose reached 66% at the end of the period. These results were obtained as part of a study about the beneficial effect of maslinic acid on cerebral ischemic injury, which will be discussed later.

Although the antidiabetic effect of maslinic acid has been extensively proved, little is known about the underlying mechanism of action. Liu *et al.* [62] confirmed the inhibitory activity of the triterpene on GPa (IC_50_ of 6.9 μM) using cell cultures of the hepatic cell line HepG2. More remarkably, the authors also hypothesized that maslinic acid targets the insulin signaling pathway [63], and found that incubation with the compound resulted in increased insulin receptor β (IRβ) phosphorylation [62]. Downstream events of IRβ activation include Akt phosphorylation, which in turn phosphorylates and inactivates glycogen synthase kinase 3β (GSK3β). GSK3β is a central enzyme in the regulation of glucose metabolism, since one of its targets is glycogen synthase. The lack of GSK3β activity allows glycogen synthase to be functional, thus resulting in glycogen build-up. Both Akt phosphorylation and GSK3β were increased in HepG2 cells in response to maslinic acid treatment, and the higher amount of glycogen content correlated with these findings. Interestingly, when maslinic acid was given orally to mice fed a high-fat diet, blood glucose concentration was markedly diminished at both doses (50 and 100 mg/kg). Moreover, the highest dose improved hyperinsulinemia and adiposity, and also increased hepatic glycogen [62].

All together, the results suggest that maslinic acid is a natural antidiabetic compound, which could be helpful to maintain the levels of blood glucose within the physiological range and thus contribute to the pharmacological treatment of the disease.

### 2.3. Maslinic Acid as Antioxidant and Anti-Inflammatory

The antioxidant effect of maslinic acid was first evaluated by Montilla *et al.* [64] in a model of oxidative status induced by CCl_4_, which induces lipid peroxidation. Pre-treatment of the rats once daily for 3 days with the triterpene at doses of 50 and 100 mg/kg reduced by approximately 18% plasma levels of endogenous lipid peroxides, at both doses, and by 6.5% and 19%, respectively, the susceptibility of plasma to lipid peroxidation [64]. Similarly, the triterpene isolated from the flowers of *Punica granatum* prevented the CuSO_4_-induced oxidation of rabbit plasma LDL, monitored by the formation of dienes, by 33.8% [65]. More recently, Allouche *et al.* [66] conducted an exhaustive study about the antioxidant properties of several pentacyclic triterpenic diols and acids on LDL particles isolated from human plasma. Maslinic acid not only retarded the initiation and decreased the rate of CuSO_4_-induced LDL oxidation, but also showed peroxyl radical scavenging activity and a slight metal (copper) chelating effect.

Further research has been done in macrophages, which play a role in the defensive system of the organism in response to activation by a pathogen [67]. Cells were isolated from murine peritoneum and activated with lipopolysaccharide (LPS), a compound that gives rise to a potent inflammatory response mediated by the production of cytokines, such as TNF-α, and also by reactive nitrogen and oxygen species, among others. In this study, the effect of the triterpene was tested on the synthesis of NO, superoxide and hydrogen peroxide. Although maslinic acid did not exert any direct inhibitory effects on the formation of the first two species, the compound did reduce the generation of hydrogen peroxide (IC_50_ of 46.3 μM), in a way that was similar to that of catalase. In addition, the release of the pro-inflammatory cytokines IL-6 and TNF-α was significantly reduced after treatment with maslinic acid at concentrations of 50 and 100 μM [67].

The anti-inflammatory activity of maslinic acid has been also proved in primary cortical astrocytes [68], which could be translated to a neuroprotective effect if further confirmed *in vivo*. Cells were cultured with the triterpene (0.1, 1, 10 μM) for 24 h before being exposed to LPS. The focus here was the TNF-α signaling pathway, which is in part mediated by NF-κB. As previously described, this transcription factor is found in the cytosol, retained by IκBα. Under stimulation, IκBα is phosphorylated and then the p65 subunit of the transcription factor is released, which allows its migration to the nucleus [39]. Maslinic acid not only suppressed the expression of TNF-α, but also hampered p65 translocation to the nucleus, which was correlated with a lower phosphorylation of IκBα. Additionally, the triterpene did inhibit the LPS-induced formation of NO, as well as mRNA and protein levels of iNOS and COX-2 [68].

Although several studies support the antioxidant activity of maslinic acid in terms of preventing LDL oxidation, the underlying mechanism remains to be clarified. In contrast, fewer assessments have been performed on the anti-inflammatory potential of the triterpene, but it seems to be driven by alterations in the TNF-α signaling pathway resulting in altered gene expression of enzymes involved in the inflammatory process.

### 2.4. Maslinic Acid and Cardioprotection

To date, the antitumor, antidiabetic and antioxidant effects of maslinic acid have focused the greatest attention, but other promising activities have been attributed to the triterpene, which contribute to raise the interest for this potential nutraceutical.

The protective effect of maslinic acid against cardiovascular diseases has been studied using different approaches, which include the assessment of the triterpene in controlling risk factors such as hypertension or hyperlipidemia.

On one hand, experiments with aortic rings isolated from spontaneously hypertensive rats showed that maslinic acid exerted a concentration-dependent relaxation (IC_50_ of 14.1 μM), after precontraction with phenylephrine [69]. The effect was endothelium-dependent, since the removal of the endothelium attenuated the relaxation. In order to elucidate the underlying mechanism, intact (with endothelium) aortic rings were pre-incubated with NG-nitro-L-arginine methyl ester (L-NAME), a NO synthase inhibitor. This resulted in a diminished relaxation in intact aortic rings, indicating that NO was involved in maslinic acid-induced vasodilation.

On the other hand, in rats fed a high-cholesterol diet for 30 days, the oral administration of maslinic acid (100 mg/kg) for the last two weeks resulted in a hypolipidemic effect, as evidenced by a reduction of more than 70% in serum triglycerides, total cholesterol and LDL-cholesterol [70]. The triterpene also restored the levels of the hepatic marker enzymes lactate dehydrogenase (LDH), alkaline phosphatase (ALP), aspartate aminotransferase (AST) and alanine aminotransferase (ALT). Similarly, both the glycogen content and the morphological alterations observed in hepatocytes were reversed in maslinic acid-treated animals, compared to controls.

The cardioprotective effect of maslinic acid has also been tested in isoproterenol-induced myocardial infarction in Wistar rats [71]. Animals that had been pre-treated with maslinic acid (15 mg/kg) for 7 days showed an improved serum lipid profile with significantly decreased levels of total cholesterol, triglycerides, LDL-cholesterol, VLDL-cholesterol and increased HDL-cholesterol. The activity of the cardiac marker enzymes creatine kinase (CK), ALT, AST and γ-glutamyl transferase (GGT) significantly decreased. Furthermore, the oxidative status of the animals was evaluated by measuring malondialdehyde (MDA), an indicator of lipid peroxidation, and paraoxonase (PON), an atheroprotective enzyme found in HDL particles [72]. MDA levels were significantly reduced, while the activity of PON increased remarkably in rats that had received maslinic acid, compared to non-treated animals [71].

In summary, maslinic acid, as a bioactive compound present in a wide variety of natural edible sources, may contribute to the beneficial effects ascribed to the Mediterranean diet on the prevention of cardiovascular diseases [73].

### 2.5. Maslinic Acid and Neuroprotection

A series of exhaustive studies have demonstrated that maslinic acid may confer neuroprotection in some pathological situations. In a first experiment with primary cultures of rat cortical neurons, cells were incubated with different concentrations of the triterpene (0.1, 1, 10 μM) and subjected to 1 h of oxygen-glucose deprivation followed by reoxygenation (24 h). Maslinic acid dose-dependently attenuated neuronal damage, which was evaluated through observation of morphological changes, release of lactate dehydrogenase (LDH) and neuronal viability [74], and this effect resulted from reduced activity of both caspase-9 and caspase-3. Upstream of caspases, high levels of NO might trigger apoptotic cell death [75]. This gaseous molecule is synthetized in great amounts by the inducible nitric oxide synthase (iNOS) in response to hypoxia [76], thus inhibition of this enzyme could be the mechanism underlying the protective effect of maslinic acid in oxygen-deprived cortical neurons. Qian *et al.* [74] observed that when challenged neurons were exposed to the triterpene (10 μM), the amount of NO in the culture medium was rescued to levels close to those found in normoxic conditions, which was correlated with reduced iNOS protein and mRNA levels.

In another study from the same authors, the neuroprotective effect of maslinic acid was assessed in front of glutamate-induced toxicity. Glutamate is the main excitatory neurotransmitter in the central nervous system, but excessive stimulation is associated with neuronal damage [77]. The removal of glutamate from the synaptic cleft takes place through the high-affinity transporters GLAST and GLT-1 located in astrocytes [78], thus ensuring the end of stimulation. In primary cultures of cortical neurons exposed to glutamate, maslinic acid did not exert any direct beneficial effects, since LDH release was comparable to that of vehicle-treated cells at all tested concentrations of maslinic acid (0.1, 1, 10 μM). However, a protective effect was indeed observed when neurons were cultured with conditioned medium obtained from astrocytes that had been incubated with maslinic acid (24 h) [79]. Further experiments evidenced that the triterpene dose-dependently increased the clearance of extracellular glutamate in cultures of astrocytes, and this was attributed to enhanced expression of both GLAST and GLT-1 after exposure to maslinic acid (10 μM). In a last assessment with co-cultures of astrocytes and neurons, maslinic acid significantly reversed the effects of glutamate in terms of LDH release, extracellular glutamate levels and neuron survival and morphology [79].

At this point it is convenient to recall the anti-inflammatory activity of maslinic acid in primary astrocytes, which has been described in a previous section [68]. All together, the results obtained from *in vitro* studies with primary cultures of neurons and astrocytes strongly support the hypothesis that maslinic acid exerts beneficial effects in the central nervous system, thus *in vivo* studies are the next step towards considering maslinic acid a neuroprotective agent.

Guan *et al.* [61] tested whether maslinic acid prevented brain damage after a transient ischemic episode in animals. Since hyperglycemia is a risk factor for stroke [80], streptozotocin-induced diabetic rats were given the triterpene orally at doses of 5 and 50 mg/kg for 14 days. Then, a transient middle cerebral artery occlusion was performed and the consequences of the infarction were evaluated. At both low and high doses, the triterpene decreased the infarct size in a range between 63.7% and 75.4%, depending on the dose and the time of reperfusion after the intervention (24 or 72 h). Moreover, maslinic acid treatment compensated the neurological deficits induced by the infarction, as showed by higher neurological scores recorded from animals that had received the triterpene [61].

To conclude, the recent interest for maslinic acid as a neuroprotective agent is supported not only by exhaustive *in vitro* studies on its mechanism of action but also by an *in vivo* assessment in infarcted diabetic rats. If proved in other species and pathological situations, the triterpene may be considered an adjuvant to lower the risk of occurrence of certain cerebral incidents.

### 2.6. Maslinic Acid as Antiparasitic

Historically, one of the first remarkable reports that focused the attention on the biological activities of maslinic acid was published by Xu *et al.* [17] and described the anti-HIV properties of several triterpenic acids isolated from the methanolic extract of *Geum japonicum*. Although the study did not provide mechanistic details of the inhibitory effect on HIV-1 protease, it is clearly stated that maslinic acid was the most potent compound [17]. More recently, the antibacterial activity of this triterpene was tested against different bacteria after its isolation from the methanolic extract of the leaves of *Symplocos lancifolia*. The lowest minimal inhibitory concentrations (MIC) of maslinic acid were found for *Enterococcus faecalis* (33.8 μM) and *Staphylococcus aureus* (135.4 μM) [81]. Although neither the antiviral nor the antibacterial activities of maslinic acid have been further studied exhaustively, the protective effect of the triterpene against parasitic infections has arisen much interest in recent years.

De Pablos *et al.* [82] observed that maslinic acid blocked the entrance of *Toxoplasma gondii* into Vero cells in a dose-dependent manner, with IC_50_ of 8 μM at 48 h of treatment. The underlying mechanism seemed to be the inhibitory activity of the triterpene against proteases secreted by the parasite, which are essential for the proteolytic processing of other proteins that participate in the invasion of host cells. Concretely, the gliding motility was suppressed by up to 100% by maslinic acid (50 μM). Moreover, the triterpene induced morphological alterations in the endomembrane systems of the parasite, such as a greater amount of apparently empty spaces that authors attribute to a possible collapse of the Golgi apparatus. Disruptions in external and nuclear membranes were also observed and attributed to a general blockage of protein turnover, which would hinder the functionality of those proteins necessary for the structural maintenance of the membranes. The same group evidenced the anti-parasitic effect of maslinic acid in *Gallus domesticus* chicks infected with *Eimeria tenella* [83]. The animals were fed a maslinic-acid supplemented diet (90 ppm) for 21 days, and this treatment resulted in a reduced release of oocysts in the faeces by 80.1%, being more effective than the positive control with sodium salinomycin (60 ppm). Histological evaluation of the caeca revealed that the characteristic lesions of this coccidiosis were less evident in the animals that had received maslinic acid. Furthermore, the body weight gain was significantly higher in treated animals compared not only to the positive control but also to the uninfected group, indicating that besides the anticoccidial activity, the triterpene enhanced weight gain [83].

Maslinic acid has also been found effective against different species of the genus *Plasmodium*, responsible of causing malaria. *In vitro* experiments using erythrocytes infected with *Plasmodium falciparum* demonstrated that maslinic acid (0.1−200 μM) inhibited the growth of the parasite in a dose-dependent manner [84]. At a concentration of 30 μM (close to the IC_50_), the triterpene reduced parasitaemia to 4% (compared to 8% in untreated red blood cells) and slowed down the cell cycle, since only the infective (schizonts) and immature (new rings) forms, but not the mature forms (trophozoites), were observed in the erythrocytes. However, the removal of maslinic acid from the medium permitted the infection to resume, meaning that the triterpene acts as a parasitostatic agent [84]. This effect was further confirmed *in vivo* with ICR mice infected with the lethal strain of *Plasmodium yoelii* [85]. The intraperitoneal injection of 40 mg/kg for 4 days increased the survival rate of the animals to 80%, compared to 20% found in animals without any experimental intervention, and this was associated with an arrest of the maturation of the parasite in the erythrocytes. In addition, the animals that survived the primary infection were rechallenged with an identical second infection 40 days later. Parasitaemia was monitored for the following 30 days but no parasites were detected, indicating that mice were completely protected against the parasite [85]. Further research on the mechanism of action underlying the antimalarial activity of maslinic acid showed that the compound hampers the maturation of the parasite inside the erythrocytes by inhibiting different proteins [86].

To sum up, several lines of evidence point to maslinic acid as antiparasitic and/or parasitostatic agent. Further research is needed in order to confirm its efficacy in target species, which would allow the use of maslinic acid either alone or in combination with other therapeutic strategies for the treatment of parasitoses.

### 2.7. Maslinic Acid and Growth

The growth-stimulating activity of maslinic acid has been studied in rainbow trouts (*Oncorhynchus mykiss*) [87,88], in order to determine whether it can be used as a feed additive in pond aquaculture to increase production rates. In both reports, the animals were fed a maslinic acid-enriched diet (1, 5, 25 and 250 mg/kg diet) twice daily for 225 days. At the end of the period, trouts that had received the highest dose of the triterpene reached a body weight that was almost 30% higher compared to the group fed the standard diet. While the first study focused on the consequences of maslinic acid consumption on the liver, the second assessed the effects on white muscle. Both of them found similar results in all the variables analyzed. The weight of the liver and the white muscle from animals that ingested the highest amount of the triterpene was 52.1% and 39.8% higher, respectively, compared to the corresponding control groups. Protein, DNA and RNA levels were evaluated in order to get some insight into the nature of the increased weight. Total DNA, which is indicative of hyperplasia, was remarkably higher in liver and white muscle, as well as RNA content. These findings were correlated with a stimulation of the protein-synthesis efficiency in both cases. Observation of the hepatic structure under the light and electron microscopes revealed a larger degree of cell packaging in the parenchyma of livers from animals that were fed the diet containing 250 mg/kg of maslinic acid, together with a major proportion of rough endoplasmic reticulum, greater number of mitochondria and considerable quantities of peripheral glycogen granules [87].

The latest contribution in this field aimed at identifying the differences in liver protein profile between fish fed a maslinic acid-supplemented diet and fish fed a standard diet [89]. The experimental design was similar to that followed in the above-mentioned studies, except for the animal species, which was the gilthead sea bream (*Sparus aurata*). The diet contained 100 mg/kg of the triterpene and was supplied over 210 days. The proteomic analysis of the liver revealed that the expression of 19 proteins was altered, being either up- or down-regulated. These included proteins involved in a wide range of metabolic pathways, such as glucose, sterol and amino acid metabolism, protein synthesis and folding, oxidative stress, detoxification and xenobiotic metabolism, immune system and cell proliferation [89]. Beyond the effects of the triterpene on the liver protein profile, this study provides evidence of the validity of the method to characterize the differential expression of liver proteins after a nutritional intervention.

In conclusion, maslinic acid appears to be a promising compound to stimulate growth by means of affecting protein synthesis. It remains to be investigated whether this effect also occurs in other species, being those subjected to intensive animal farming of particular interest. If proved, maslinic acid may be considered a natural growth promoter and thus constitute another alternative to the use of hormones or antibiotics to increase production rates.

### 2.8. Other Biological Activities

To date, the previously described health-enhancing properties of maslinic acid have focused the major attention, as evidenced by the fact that each of them has been addressed by several studies. However, maslinic acid has also been attributed a variety of other biological effects, which include the inhibition of elastase [90] and tyrosinase [91] * in vitro*, the suppression of osteoclastogenesis in cell cultures and the prevention of ovariectomy-induced bone loss in mice [92], antinociceptive and antiallodynic effects in different pain models in mice [93], and the ability to alter the structural properties of biological membranes [94].

## 3. Conclusion and Future Prospects

Maslinic acid is a natural pentacyclic triterpene present in a variety of plant species, many of them being common ingredients of plant-based dietary patterns, such as the Mediterranean diet. In recent years, a number of studies assessing its biological effects have raised interest in this compound. These include not only health-enhancing properties, such as cardioprotective or neuroprotective, but also a therapeutic potential that may help in the treatment of several disorders, such as cancer, diabetes or parasitoses. However, the amount of maslinic acid in natural edible sources is low, and data about its pharmacokinetics, which we are currently assessing in our laboratory, show that the triterpene has a poor oral bioavailability. From this it would appear that dietary maslinic acid is not sufficient to reach effective concentrations in target organs, thus the compound should be supplied in pure form, *i.e.* as a nutraceutical. Nevertheless, maslinic acid is in the spotlight of research on this field. Further studies will surely provide new mechanisms of action to explain the effects already described or even widen the spectrum of biological activities of this pentacyclic triterpene.
